# Brief Report: Severe Pneumonitis After Combined Thoracic Radiotherapy and Osimertinib

**DOI:** 10.1016/j.jtocrr.2023.100468

**Published:** 2023-02-02

**Authors:** Clayton P. Smith, Michael Xiang, Stephanie M. Yoon, Alan Lee, Dan Ruan, Jonathan W. Goldman, Amy L. Cummings, Aaron Lisberg, Edward B. Garon, Drew Moghanaki

**Affiliations:** a Department of Radiation Oncology, David Geffen School of Medicine, University of California, Los Angeles, Los Angeles, California; b Division of Hematology and Oncology, Department of Medicine, David Geffen School of Medicine, University of California, Los Angeles, Los Angeles, California

**Keywords:** Pneumonitis, Radiation therapy, Osimertinib, EGFR-mutated NSCLC

## Abstract

**Introduction:**

Osimertinib is an effective treatment for metastatic NSCLC. Occasionally, thoracic radiation therapy (TRT) is delivered to patients receiving osimertinib to treat residual or progressing pulmonary tumors. Anecdotal reports suggest that the delivery of TRT in combination with osimertinib may be associated with a high risk of severe pneumonitis.

**Methods:**

A retrospective study was performed at a single academic medical center in the United States to investigate the incidence of severe pneumonitis among patients treated with combined TRT and osimertinib between June 2016 and December 2021. Baseline patient characteristics, tumor size and location, and dosimetric parameters were evaluated. The highest grade of radiation pneumonitis that developed within 6 months of treatment was scored in accordance with the Common Terminology Criteria for Adverse Events version 5.0.

**Results:**

A total of 16 patients were identified who were treated with combined TRT and osimertinib. All had a diagnosis of metastatic NSCLC. Treatment-related grade greater than or equal to 2 pneumonitis developed in 56%, grade greater than or equal to 3 in 37.5%, and grade 4 in 6.3%; no patient developed grade 5 pneumonitis. Median time to any-grade pneumonitis was 29 days (1–84 d); all patients had symptom resolution with expectant management or oral steroid therapies. All patients discovered to have grade greater than or equal to 3 pneumonitis (n = 6) received TRT to tumors located within 2 cm of the proximal bronchial tree, including tumors abutting the proximal bronchial tree (n = 2) and within the mediastinum (n = 1).

**Conclusions:**

The combination of TRT with osimertinib was associated with a high rate of severe pneumonitis that required oral steroid medications. Larger studies are needed to validate these findings and to understand the clinical and treatment factors that influence this risk and how they can be mitigated.

## Introduction

EGFR tyrosine kinase inhibitors (TKIs) have improved outcomes for patients with NSCLC.^1^, ^2^, ^3^ The third-generation EGFR TKI osimertinib was found to have survival benefits than first-generation agents gefitinib and erlotinib, and it is now a standard of care for metastatic NSCLC.^4^ Depending on clinical scenarios, thoracic radiation therapy (TRT) may be prescribed to oligoremnant or oligoprogressive tumors in the lungs to improve tumor control. Randomized trials are ongoing to evaluate the value of radiation therapy in patients with oligometastatic NSCLC after initial response to a TKI (NCT03256981).

Although the role of TRT in patients with oligometastatic NSCLC seems promising, there is a paucity of data on its safety when combined with osimertinib. It is known that osimertinib causes interstitial lung disease in approximately 4% of patients.^5^ TRT can also cause severe pneumonitis in 3% to 5% of patients with oligometastatic NSCLC.^6^ The first reported case of severe pneumonitis associated with combined TRT and osimertinib was from Tenon Hospital in Paris by Sanchis-Borja et al.^7^ in 2019. A case series (n = 11) from Shandong Cancer Hospital by Jia et al.^8^ in 2020 reported a grade greater than or equal to 3 pneumonitis rate of 54.5% and one fatal complication. Given the limited data available, an independent review was performed to evaluate the rate of severe pneumonitis with combined TRT and osimertinib at our institution.

## Materials and Methods

A retrospective study was performed to identify patients who were treated with combined TRT and osimertinib between June 2016 and December 2021 at a single academic medical center. Patients were excluded if they had a history of interstitial lung disease. This work was completed under a waiver of informed consent.

Baseline patient characteristics, tumor size and location, and dosimetric parameters were recorded. Tumor location was coded as central (within 1–2 cm of the proximal bronchial tree), ultracentral (within 1 cm of the proximal bronchial tree), mediastinal (within the mediastinum), or peripheral (all other lung parenchymal locations). All patients underwent stereotactic body radiation therapy (SBRT) or intensity-modulated radiation therapy with photon therapy. TRT treatment plans were developed using four-dimensional computed tomography simulations and daily image guidance using cone beam computed tomography. Patients received osimertinib orally at 80 mg daily until disease progression was deemed uncontrollable by a medical oncologist or until the patient developed intolerable toxicities.

The primary outcome of interest was severe grade greater than or equal to 3 pneumonitis within 6 months of TRT based on documentation in the electronic health record in accordance with the Common Terminology Criteria for Adverse Events version 5.0.

## Results

A total of 16 patients were treated with combined TRT and osimertinib over the study time period; the median age was 70 years and all had a diagnosis of metastatic NSCLC (Table 1). The TRT treatment site was peripheral in four (25%), central in four (25%), ultracentral in seven (44%), and mediastinal in one (6%). TRT was delivered with a median prescription dose of 50 Gy (11–65 Gy) in a median of 7.5 fractions (1–15 fractions); the median duration of TRT was 10 days (1–22 d).

**Table 1:** Patient and Treatment Characteristics

Patient	Age (y)	Pulmonary Comorbidity	Smoking Status at TRT	Pack-Year Smoking History	Duration of Osimertinib Before TRT (mo)	Osimertinib Sequence With TRT	Tumor Location	Tumor Volume (mL)	TRT Dose Prescription	Volume of Ipsilateral Lung ≥ 20 Gy (mL)	Pneumonitis Grade	Time to Symptomatic Pneumonitis From TRT Start Date (d)
1	63	None	Never smoker	Unknown	7	Concurrent	Peripheral	6.4	18 Gy × 3	45	0	-
2	69	None	Prior smoker	30	21	Concurrent	Peripheral	1.6	10 Gy × 5	21	0	-
3	85	None	Never smoker	Unknown	5	Concurrent	Peripheral	0.5	18 Gy × 3	27	2	69
4	95	None	Never smoker	Unknown	28	Concurrent	Peripheral	24.9	11 Gy × 1	0	2	4
5	72	None	Never smoker	Unknown	13	Concurrent	Central	9.8	18 Gy × 3	162	0	-
6	82	None	Prior smoker	Unknown	<1	Concurrent	Central	14.0	6.5 Gy × 10	154	3	42
7	76	Asthma	Never smoker	Unknown	10	Concurrent	Central	6.8	12.5 Gy × 4	153	3	61
8	70	Emphysema, asthma	Current smoker	Unknown	6	Concurrent	Central	0.7	18 Gy × 3	63	4	8
9	54	None	Never smoker	Unknown	18	Concurrent	Ultracentral	9.2	4 Gy × 15	136	0	-
10	51	None	Prior smoker	5	2	Held 24 h before and after TRT	Ultracentral	27.9	5 Gy × 10	356	0	-
11	77	None	Never smoker	Unknown	<1	Held 48 h before and after TRT	Ultracentral	50.8	4 Gy × 10	384	0	-
12	72	None	Prior smoker	Unknown	4	Concurrent	Ultracentral	428	3 Gy × 10	154	0	-
13	60	None	Prior smoker	Unknown	5	Concurrent	Ultracentral	43.9	8 Gy × 5	226	2	11
14	63	None	Never smoker	Unknown	16	Concurrent	Ultracentral	3.6	6.5 Gy × 10	111	3	97
15	63	None	Never smoker	Unknown	12	Concurrent	Ultracentral	23.8	4 Gy × 10	218	3	45
16^a^	42	None	Prior smoker	**1.5**	<1	Concurrent	Mediastinal	-	2.5 Gy × 11	-	3	38

RT, radiation therapy; TRT, thoracic radiation therapy.

a TRT was discontinued after 11 fractions due to development of intolerable esophagitis; comprehensive treatment records were unavailable to ascertain tumor volume and volume of ipsilateral lung receiving ≥20 Gy. Red indicates patients who developed grade 3 or higher radiation pneumonitis.

The median follow-up time was 13 months (1–61 mo). Nine patients developed grade greater than or equal to 2 pneumonitis (56%) with a median time to onset of 29 days (1–84 d); the severity of pneumonitis was grade greater than or equal to 3 in six patients (37.5%). A single patient with a history of chronic obstructive pulmonary disease requiring multiple previous hospitalizations developed grade 4 pneumonitis after TRT; no patients were identified with grade 5 pneumonitis.

**Figure 1:**
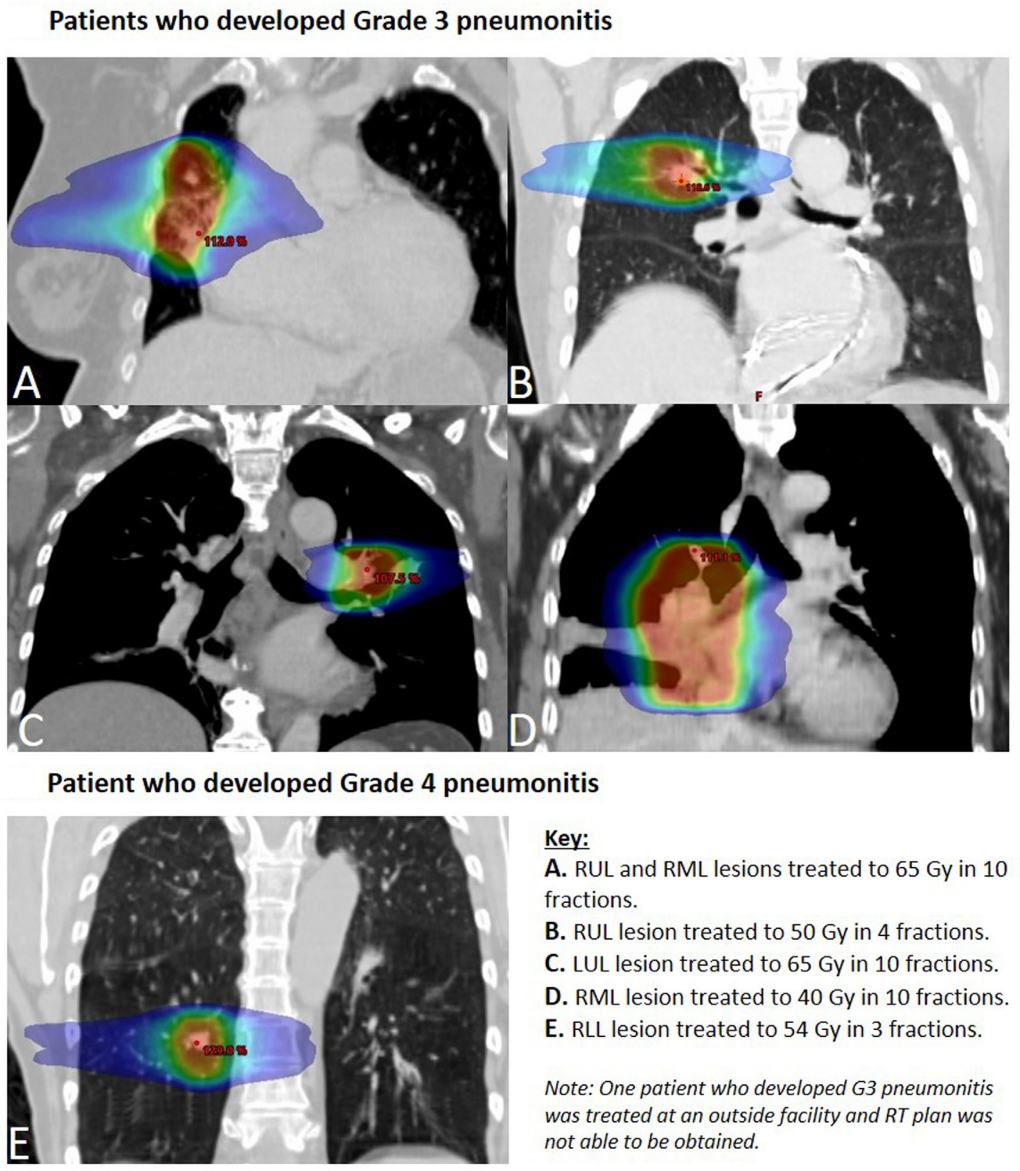
Thoracic radiation therapy treatment plans for patients who developed grade 3 or higher pneumonitis.

As displayed in Figure 1, TRT plans resulting in grade greater than or equal to 3 pneumonitis targeted tumors that were central in three (50%), ultracentral in two (33%), and mediastinal in one (17%). The TRT prescriptions were SBRT in three to four fractions for two patients (33%) and consisted of a protracted course of hypofractionated radiation therapy in 10 to 11 fractions for four patients (66.6%). Two of 16 patients withheld osimertinib 24 to 48 hours before and after TRT, both of whom had tumors in an ultracentral location, and neither was found to have developed pneumonitis. Severe pneumonitis was not associated with the use of SBRT versus hypofractionated TRT or mean lung dose. All patients developing grade 2 pneumonitis had resolution of symptoms with expectant management; patients developing grade 3 to 4 pneumonitis had symptom resolution with oral steroid therapies.

## Discussion

To best of our knowledge, this is the largest study to date investigating the risk of severe pneumonitis after combined TRT and osimertinib in patients with metastatic NSCLC. It is also the first report on the topic from a North American institution. The findings support previously reported safety concerns whenever TRT is delivered to patients receiving osimertinib.

EGFR TKIs are generally safe with a severe pneumonitis risk of less than 5%.^4^^,^^9^^,^^10^ Reports published as early as 2014 have identified that the combination of curative-intent TRT with first-generation TKIs increases the risk up to 23% in patients with stage III NSCLC.^9^^,^^11^^,^^12^ More recent reports, including this one, reveal the risk of severe pneumonitis may be as high as 45% with the combination of SBRT and hypofractionated TRT with the third-generation osimertinib for patients with stage IV NSCLC.

It deserves emphasis that severe pneumonitis events in this report were limited to patients who received TRT for tumors located within 2 cm of the proximal bronchial tree. Previous studies have revealed that SBRT targeting this area is independently associated with a high risk of severe pneumonitis.^13^ Nevertheless, the TRT doses delivered with osimertinib in this report were moderate in comparison to previous studies and thus were unlikely to have been the sole contributor to pulmonary injury. The small size of this case series limited the ability to analyze contributions of TRT prescriptions independent of its possible interactions with osimertinib.

### Limitations

This is a small case series of patients at a single institution who were referred for TRT from a broader cohort of patients with EGFR-mutated NSCLC. Although these data are important to the growing body of literature in TKI-based therapy combined with TRT, this group of patients is very diverse and dissimilar, and thus it can be challenging to make meaningful conclusions. Therefore, the generalizability of the reported findings to broader populations cannot be known without a larger study.

## Conclusions

The combination of TRT and osimertinib was associated with a high rate of severe pneumonitis limited to patients with pulmonary tumors located within 2 cm of the proximal bronchial tree. These data raise questions about safety issues that are not currently listed in treatment guidelines, although it should be noted that meaningful conclusions are difficult to make based on a small retrospective case series study. Further research is indicated to validate these findings in larger cohorts to determine whether patient selection and treatment factors can be modified to mitigate this risk.

## CRediT Authorship Contribution Statement

**Clayton P. Smith**: Conceptualization, Data curation, Formal analysis, Investigation, Methodology, Project administration, Roles/Writing—Original draft, Writing—Review and editing.

**Michael H. Xiang:** Data curation, Formal analysis, Writing—Review and editing.

**Stephanie M. Yoon:** Methodology, Writing—Review and editing.

**Alan Lee:** Writing—Review and editing.

**Dan Ruan:** Data curation.

**Jonathan W. Goldman:** Writing—Review and editing.

**Amy L. Cummings:** Writing—Review and editing.

**Aaron Lisberg:** Writing—Review and editing.

**Edward B. Garon:** Writing—Review and editing.

**Drew Moghanaki:** Conceptualization, Data curation, Formal analysis, Investigation, Methodology, Project administration, Roles/Writing—Original draft, Writing—Review and editing.

## References

[1] Wu Y.L., Zhou C., Liam C.K. (2015). First-line erlotinib versus gemcitabine/cisplatin in patients with advanced EGFR mutation-positive non-small-cell lung cancer: analyses from the phase III, randomized, open-label, ENSURE study. Ann Oncol.

[2] Rosell R., Carcereny E., Gervais R. (2012). Erlotinib versus standard chemotherapy as first-line treatment for European patients with advanced EGFR mutation-positive non-small-cell lung cancer (EURTAC): a multicentre, open-label, randomised phase 3 trial. Lancet Oncol.

[3] Zhou C., Wu Y.L., Chen G. (2011). Erlotinib versus chemotherapy as first-line treatment for patients with advanced EGFR mutation-positive non-small-cell lung cancer (OPTIMAL, CTONG-0802): a multicentre, open-label, randomised, phase 3 study. Lancet Oncol.

[4] Ramalingam S.S., Vansteenkiste J., Planchard D. (2020). Overall survival with osimertinib in untreated, *EGFR*-mutated advanced NSCLC. N Engl J Med.

[5] Soria J.C., Ohe Y., Vansteenkiste J. (2018). Osimertinib in untreated *EGFR*-mutated advanced non–small-cell lung cancer. N Engl J Med.

[6] Siva S., Kron T., Bressel M. (2016). A randomised phase II trial of stereotactic ablative fractionated radiotherapy versus radiosurgery for oligometastatic neoplasia to the lung (TROG 13.01 SAFRON II). BMC Cancer.

[7] Sanchis-Borja M., Parrot A., Sroussi D., Rivin Del Campo E., Fallet V., Cadranel J. (2019). Dramatic radiation recall pneumonitis induced by osimertinib after palliative thoracic radiotherapy for lung cancer. J Thorac Oncol.

[8] Jia W., Guo H., Jing W. (2020). An especially high rate of radiation pneumonitis observed in patients treated with thoracic radiotherapy and simultaneous osimertinib. Radiother Oncol J Eur Soc Ther Radiol Oncol.

[9] Wang X.S., Bai Y.F., Verma V. (2022). Randomized trial of first-line tyrosine kinase inhibitor with or without radiotherapy for synchronous oligometastatic EGFR-mutated NSCLC. J Natl Cancer Inst.

[10] Wu Y.L., Cheng Y., Zhou X. (2017). Dacomitinib versus gefitinib as first-line treatment for patients with EGFR-mutation-positive non-small-cell lung cancer (ARCHER 1050): a randomised, open-label, phase 3 trial. Lancet Oncol.

[11] Zhuang H., Yuan Z., Chang J.Y. (2014). Radiation pneumonitis in patients with non–small-cell lung cancer treated with erlotinib concurrent with thoracic radiotherapy. J Thorac Oncol.

[12] Xu K., Liang J., Zhang T. (2021). Clinical outcomes and radiation pneumonitis after concurrent EGFR -tyrosine kinase inhibitors and radiotherapy for unresectable stage III non-small cell lung cancer. Thorac Cancer.

[13] Swaminath A., Ritter T., Louie A.V. (2022). Performing SBRT in the fly-with-caution zone: are we heeding the advice of Daedalus?. Int J Radiat Oncol Biol Phys.

